# Hsa_circ_0000190 Promotes NSCLC Cell Resistance to Cisplatin via the Modulation of the miR-1253/IL-6 Axis

**DOI:** 10.1155/2024/6647810

**Published:** 2024-02-26

**Authors:** Hua He, Tian Li

**Affiliations:** Department of Respiratory, Nanjing Chest Hospital, Affiliated Nanjing Brain Hospital, Nanjing Medical University, No. 215, Guangzhou Road, Nanjing 210029, China

## Abstract

**Background:**

This study explored the mechanistic basis for nonsmall cell lung cancer (NSCLC) cisplatin (DDP) treatment resistance in an effort to define effective approaches to abrogating the emergence of such chemoresistance.

**Methods:**

Analyses of NSCLC expression of hsa_circ_0000190, miR-1253, and interleukin 6 (IL-6) were conducted via a quantitative real-time polymerase chain reaction (qPCR) approach, while the ability of these tumor cells to resist DDP treatment was evaluated with a CCK-8 assay. Interactions between different RNA molecules were assessed using both RNA immunoprecipitation and dual-luciferase reporter assays.

**Results:**

NSCLC cell lines and tissues resistant to DDP were found to express higher levels of hsa_circ_0000190, and knocking down this circRNA in NSCLC cells was associated with greater sensitivity to DDP exposure. Further research identified miR-1253 as a hsa_circ_0000190 target, with the ability of hsa_circ_0000190 knockdown to restore DDP sensitivity being largely attributable to the ability of this circRNA to suppress miR-1253 activity. IL-6 was identified as a major miR-1253 target in this context, with miR-1253 regulating chemoresistance in NSCLC cells in part by preventing IL-6 upregulation.

**Conclusion:**

Together, these data suggest that hsa_circ_0000190 can promote DDP chemoresistance in NSCLC cells through its ability to modulate miR-1253/IL-6 axis activity, highlighting a novel pathway that can be targeted in an effort to guide the more effective diagnosis and management of DDP-resistant tumors.

## 1. Introduction

Nonsmall cell lung cancer (NSCLC) cases make up an estimated 85% of all primary lung tumor diagnoses [1]. NSCLC patients are often treated with a chemotherapeutic regimen that includes cisplatin (DDP) [2], but the emergence of DDP resistance ultimately constrains the efficacy of this interventional strategy and contributes to poor prognostic outcomes [3]. Research focused on elucidating the mechanistic basis for the emergence of chemoresistance in NSCLC thus has the potential to provide a novel means of restoring therapeutic sensitivity to target tumors, ultimately contributing to better antitumor efficacy.

Circular RNAs (circRNAs) are a series of endogenously encoded RNA transcripts that form a closed loop as a result of the covalent linkage of the 3′ and 5′ ends of exonic sequences [4, 5]. These circRNAs have increasingly been codified as key regulators of a diverse array of oncogenic processes [6]. Hsa_circRNA_104348, for example, is capable of targeting the miR-187-3p/rhotekin 2 (RTKN2) axis and driving the activation of Wnt/*β*-catenin signaling in a manner conducive to hepatocellular carcinoma progression [7]. In NSCLC, circRNA_0000429 has been reported as a molecular sponge capable of sequestering miR-1197, thereby modulating the expression of MAP-kinase activating death domain (MADD) [8]. Hsa_circ_0000190 can also promote NSCLC tumor growth by inducing soluble PD-L1 upregulation such that these tumors can better evade immune-mediated elimination [9]. Hsa_circ_0000190 can similarly regulate epidermal growth factor receptor (EGFR)/extracellular regulated protein kinases (ERK) pathway activity in a manner beneficial to NSCLC tumor cells [10]. Despite its important tumorigenic role in this form of lung cancer, however, no publications to date have documented the impact of hsa_circ_0000190 on the emergence of DDP resistance in NSCLC.

Small single-stranded noncoding transcripts known as miRNAs are capable of regulating the vast majority of known biological processes [11], doing so by pairing with complementary target mRNA 3′ untranslated region (UTR) sequences and thereby reducing the translation of these transcripts [12]. Many tumors reportedly exhibit the dysregulation of miR-1253, and this miRNA has been reported to be a target of several circRNAs, functioning in a tumor suppressor-like manner [13–15]. Little research to date, however, has focused on the association between hsa_circ_0000190 and miR-1253 in NSCLC. IL-6 is a cytokine that can drive NF-*κ*B-mediated TIM4 upregulation and consequent NSCLC cell metastatic progression [16], in addition to inducing the phosphorylation of BECN1 so as to modulate chemoresistance and autophagic activity [17].

In this study, an in-depth analysis was conducted of the potential role of the hsa_circ_0000190/miR-1253/IL-6 axis as a regulator of DDP resistance in NSCLC. Following initial analyses of hsa_circ_0000190 expression patterns in NSCLC, the mechanistic role of this circRNA as a modulator of NSCLC cell malignancy and chemoresistance was assessed.

## 2. Materials and Methods

### 2.1. Clinical Samples

In total, 136 paired NSCLC tumor and normal tissue samples were harvested from patients at Nanjing Chest Hospital, Affiliated Nanjing Brain Hospital, Nanjing Medical University. The patients from whom these samples had been harvested were separated into two subgroups based on their DDP sensitivity, including DDP-sensitive patients (*n* = 63) and DDP-resistant patients (*n* = 73). The Ethical Committee of Nanjing Chest Hospital, Affiliated Nanjing Brain Hospital, Nanjing Medical University (No. 000128) approved all work using human samples, and all patients gave written consent. The patient characteristics of hsa_circ_0000190 were showed as Table 1.

### 2.2. Cell Culture

The A549 and H460 human NSCLC cell lines, control HBE1 cells, and 293T cells were from the BeNa culture collection (Beijing, China). NSCLC cells resistant to DDP (Sigma, MO, USA) were established as reported previously to generate the A549/DDP and H460/DDP cell lines. All cells were cultured in RPMI-1640 (Hyclone, UT, USA) containing 10% fetal bovine serum (FBS; Gibco, CA, USA) and penicillin/streptomycin (Sigma) in a 5% CO_2_ incubator at 37°C.

### 2.3. Quantitative Real-Time Polymerase Chain Reaction (qPCR)

At 48 hr posttransfection, Trizol (Invitrogen, CA, USA) was used to extract total cellular RNA. When analyzing hsa_circ_0000190 levels in samples, linear transcripts were eliminated through RNase (Epicentre, WI, USA) treatment. A First Strand cDNA Synthesis Kit (Toyobo, Tokyo, Japan) and a MicroRNA Reverse Transcription Kit (Applied Biosystems, CA, USA) were used for cDNA synthesis. All qPCR reactions were performed using reaction wells containing equal amounts of primers, cDNA, and reagents from the SYBR Premix Ex Taq Kit (Qiagen, CA, USA). The 2^−∆∆Ct^ method was used to assess relative expression levels, with GAPDH and U6 serving as normalization controls. The primer sequences for QPCR are as follows: Hsa_circ_0000190, 5′GATCCAACAGAAATACACAATCGAGGG3′ and 5′GCAGTAATACAGTGACAATGGTATGGC3′; miR-1253, 5′GCTGTAACAGCGGCGGAACTCC3′ and 5′ATCCGCAGGAGTGTCCGAG3′; IL-6, 5′ GCTGCTCCTGGTGATGACTTC3′ and 5′GGTGGTGTCATTTTTGAAATCTTCT3′; GAPDH, 5′GGATATTGTTGCCATCAATGACC3′ and 5′AGCCTTCTCCATGGTGGTGAAGA3′; U6, 5′GCTTCGGCAGCACATATACTAAAAT3′ and 5′CGCTTCACGAATTTGCGTGTCAT3′.

### 2.4. RNase R and Actinomycin D Treatment

Hsa_circ_0000190-containing samples were treated using RNase R (Applied BIOLOGICAL Materials, Vancouver, Canada) for 20 min at a dose of 100 *μ*g/mL with subsequent qPCR analysis in order to confirm the circular nature of this transcript. To confirm the stability of this circRNA transcript, NSCLC cells were treated using Actinomycin D (Sigma) at a dose of 2 mg/mL, with transcript levels subsequently being analyzed via qPCR.

### 2.5. Subcellular Localization

A PARIS™ Kit Protein and RNA Isolation system (Thermo Fisher Scientific, MA, USA) was used based on provided directions to isolate RNA from the nuclear and cytosolic fractions.

### 2.6. Cell Transfection

Lipofectamine 2000 (Invitrogen) was used for all transfection experiments using si-hsa_circ_0000190, si-NC, miR-1253 mimics, miR-NC constructs, miR-1253 inhibitors (anti-miR-1253), miR-NC inhibitor (anti-miR-NC) control constructs, pcDNA3.1-IL-6, or pcDNA3.1, all of which were produced by GenePharma (Shanghai, China) and Ribobio (Guangzhou, China). At 8 hr posttransfection, media was exchanged for fresh culture media.

### 2.7. CCK-8 Assay

The resistance of NSCLC cells to DDP administration was evaluated with a CCK-8 kit (Beyotime, Jiangsu, China). In brief, at 48 hr posttransfection with appropriate constructs or plasmids of interest, cells were plated in 96-well plates. Cells were then treated using various DDP doses (0, 0.01, 0.1, 0.5, 1, 5, 10, or 20 *μ*g/mL) following overnight incubation, and 10 *μ*L/well of CCK-8 reagent was added for 4 hr. Absorbance at 450 nm was then measured as a means of quantifying the IC50 values for DDP.

### 2.8. Dual-Luciferase Reporter Assay

The Circular RNA Interactome (https://circinteractome.nia.nih.gov/index.html) and circBank (http://www.circbank.cn) databases were used to predict possible miRNA targets of hsa_circ_0000190, while TargetScan was used to identify possible miR-1253 mRNA targets. To validate these interactions, the wild-type (WT) hsa_circ_0000190 or IL-6 sequences harboring miR-1253 complementarity or mutated (MUT) versions of these sequences were introduced into the pmirGLO vector (Promega, WI, USA). The resultant plasmids (hsa_circ_0000190 WT, hsa_circ_0000190 MUT, IL-6 3′UTR WT, and IL-6 3′UTR MUT) were transfected into 293T cells together with miR-1253 or miR-NC constructs as appropriate. A dual-luciferase reporter assay kit (Promega) was used at 24 hr posttransfection to quantify luciferase activity in these samples.

### 2.9. RNA Immunoprecipitation (RIP)

To confirm the ability of miR-1253 and hsa_circ_0000190 to interact directly with one another, the EZ-Magna RIP™ RNA-Binding Protein Immunoprecipitation Kit (Millipore) kit was used based on provided instruction. Lysis buffer supplemented with RNase inhibitor (Millipore) was used for the initial preparation of cell lysates, which were subsequently incubated with Argonaute2 (Ago2; Millipore) or Immunoglobulin G (IgG; Millipore) antibody-coated magnetic beads. A qPCR approach was subsequently used to quantify RNA enrichment.

### 2.10. Statistical Analysis

GraphPad Prism 8.0 (GraphPad, CA, USA) was used to conduct all analyses, and data are presented as means ± standard deviations (SD). Results were compared with Student's *t* tests and one-way ANOVAs. Spearman's correlation analyses were used to assess linear relationships among variables. All experiments were conducted in triplicate, with *P* < 0.05 as the cutoff used to define statistical significance.

## 3. Results

### 3.1. DDP-Resistant NSCLC Tissues and Cells Lines Exhibit Hsa_circ_0000190 Upregulation

**Figure 1:**
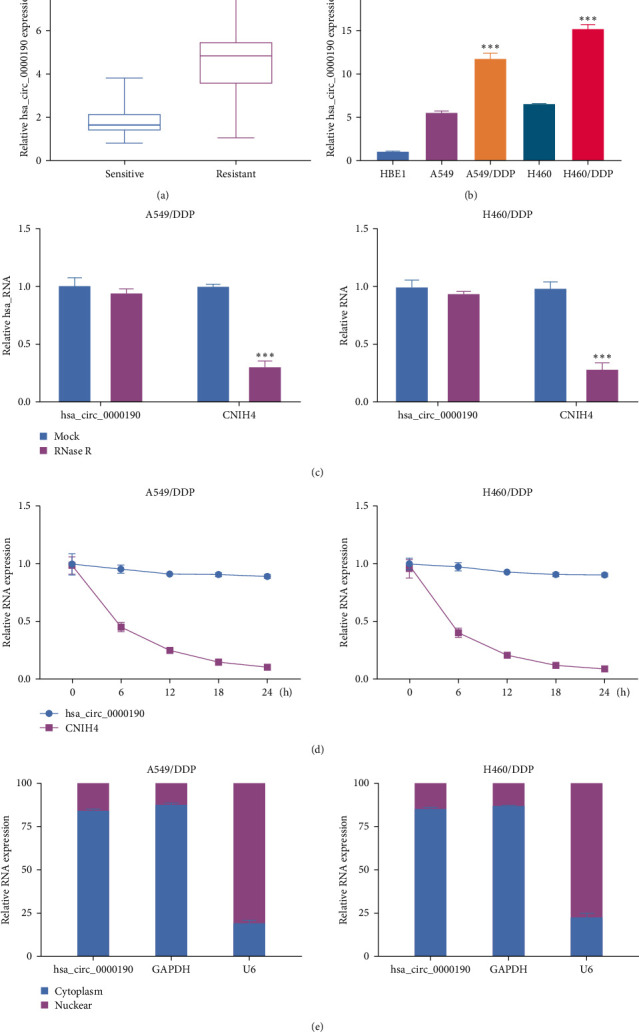
DDP-resistant NSCLC tissues and cells lines exhibit hsa_circ_0000190 upregulation. (a) Hsa_circ_0000190 levels were assessed via qPCR in NSCLC tumor tissues harvested from DDP-resistant (*n* = 73) and DDP-sensitive (*n* = 63) patients. (b) Hsa_circ_0000190 levels were analyzed in NSCLC cell lines, DDP-resistant NSCLC cell lines, and control HBE1 cells via qPCR. (c) The closed covalent loop nature of hsa_circ_0000190 was evaluated through treatment with RNase R, using the linear CNIH4 transcript as a control. (d) Actinomycin D treatment was used to inhibit transcription in order to evaluate hsa_circ_0000190 stability, using the linear CNIH4 transcript as a control. (e) A PARIS™ Kit Protein and RNA Isolation system was used to evaluate the subcellular localization of hsa_circ_0000190.  ^*∗∗∗*^*P* < 0.001.

To initially probe the potential link between hsa_circ_0000190 and the emergence of chemoresistance to DDP in NSCLC, tissue samples from DDP-sensitive and DDP-resistant patients were collected and the levels of hsa_circ_0000190 therein were quantified by qPCR. This approach revealed the significant upregulation of hsa_circ_0000190 in DDP-resistant tumor tissues as compared to DDP-sensitive samples (Figure 1(a)). Hsa_circ_0000190 levels in NSCLC cell lines were also altered relative to HBE1 control cells, and the expression of this circRNA was further enhanced in DDP-resistant sublines derived from these NSCLC cells (Figure 1(b)), supporting the observed data derived from human tissues. The DDP of IC50 was enhanced in DDP resistant cells relative to normal cells (Figure S1). Treatment of RNA extracts from these cells revealed that the exonuclease RNase R was not able to effectively digest hsa_circ_0000190, consistent with its covalent closed loop structure (Figure 1(c)). When transcription was inhibited using actinomycin D, hsa_circ_0000190 also exhibited stability superior to that of the linear CNIH4 mRNA transcript (Figure 1(d)). Subcellular localization analyses of hsa_circ_0000190 also revealed that it was primarily localized in the cytosol of NSCLC cells (Figure 1(e)), suggesting that it may function as a sponge capable of sequestering target miRNAs, providing a possible mechanism whereby this circRNA may shape the chemoresistance of NSCLC cells.

### 3.2. Silencing Hsa_circ_0000190 Sensitizes Chemoresistant NSCLC Cells to DDP

**Figure 2:**
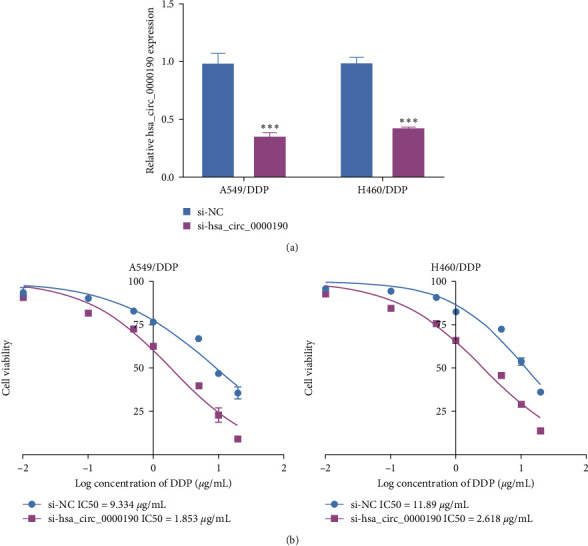
Silencing hsa_circ_0000190 sensitizes chemoresistant NSCLC cells to DDP. (a) Si-hsa_circ_0000190 transfection efficiency was assessed via qPCR. (b) A CCK-8 assay approach was used to quantify DDP IC50 values.  ^*∗∗∗*^*P* < 0.001.

Successful hsa_circ_0000190 knockdown was confirmed via qPCR in treated NSCLC cell lines (Figure 2(a)), and CNIH4 mRNA expression was no difference between hsa_circ_0000190 knockdown cell and control cells (Figure S2). The silencing of this circRNA resulted in significant decreases in DDP IC50 values for both A549/DDP and H460/DDP cells (Figure 2(b)) and overexpression of hsa_circ_0000190 increased DDP IC50 values for both A549/DDP and H460/DDP cells (Figure S3), suggesting that hsa_circ_0000190 can enhance NSCLC cell resistance to DDP treatment in a manner.

### 3.3. Hsa_circ_0000190 Functions as a Molecular Sponge Capable of Sequestering miR-1253

**Figure 3:**
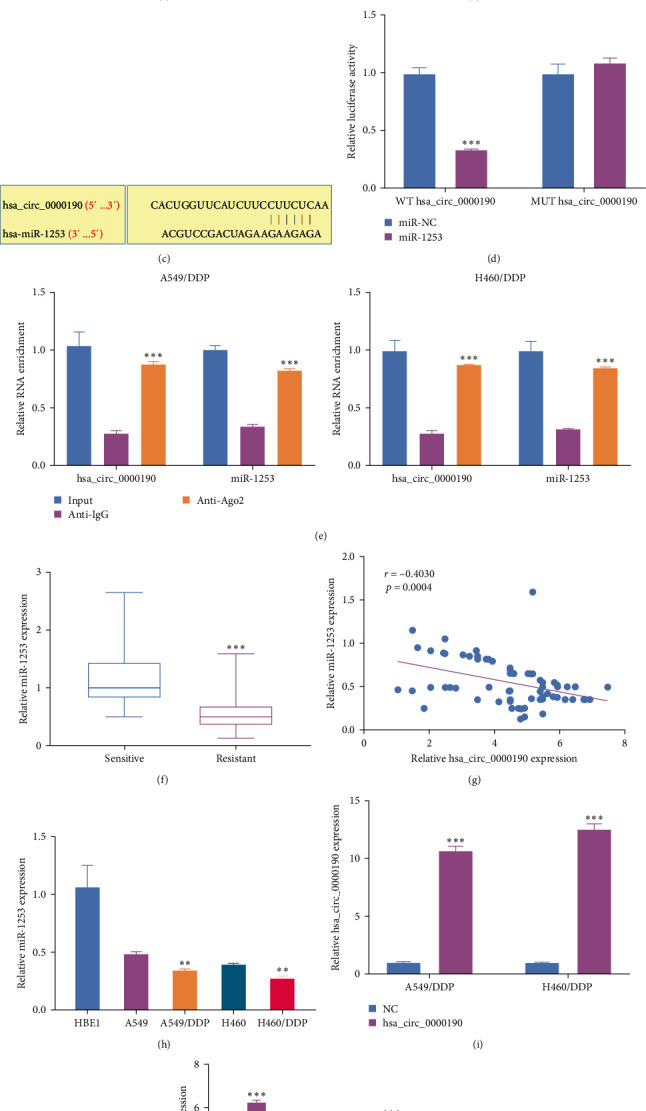
Hsa_circ_0000190 functions as a molecular sponge capable of sequestering miR-1253. (a) qPCR was used to assess the levels of nine miRNA candidates in NSCLC patient tumors and paracancerous tissues. (b) qPCR was used to assess miR-1253 levels in A549/DDP and H460/DDP cells following si-NC or si-hsa_circ_0000190 transfection. (c) The identified sequence overlap between hsa_circ_0000190 and miR-1253. (d, e) The ability of hsa_circ_0000190 and miR-1253 to interact was assessed through dual-luciferase reporter assays (d) and RIP assays (e). (f) miR-1253 levels were assessed via qPCR in NSCLC tumor tissues harvested from DDP-resistant (*n* = 73) and DDP-sensitive (*n* = 63) patients. (g) Spearman's correlations were used to assess linear relationships between hsa_circ_0000190 and miR-1253 levels in DDP-resistant NSCLC tissues. (h) qPCR was used to detect miR-1253 in the indicated NSCLC and control cell lines. (i) qPCR was used to quantify hsa_circ_0000190 transfection efficiency. (j) miR-1253 expression in A549/DDP and H460/DDP was quantified via qPCR following vector, hsa_circ_0000190, si-NC, or si-hsa_circ_0000190 transfection.  ^*∗∗∗*^*P* < 0.001 and  ^*∗∗*^*P* < 0.01.

In total, the circular RNA interactome identified 9 putative hsa_circ_0000190 target miRNAs. Of these, only miR-1253 was downregulated in NSCLC tissues, whereas miR-1252, miR-1299, miR-1825, and miR-382 were upregulated, and no differences in miR-142-5p, miR-516b, miR-580, or miR-767-5p were noted (Figure 3(a)). The knocking down of hsa_circ_0000190 resulted in miR-1253 upregulation (Figure 3(b)), and a miR-1253 binding site was present within hsa_circ_0000190 (Figure 3(c)). RIP and dual-luciferase reporter assays were next used to probe the ability of these two transcripts to interact with one another. Following miR-1253 mimic transfection, WT hsa_circ_0000190 luciferase reporter activity was suppressed, whereas the same was not true for the MUT reporter, confirming a direct targeting relationship between hsa_circ_0000190 and miR-1253 within 293T cells (Figure 3(d)). An Ago2 antibody preferentially precipitated both hsa_circ_0000190 and miR-1253 relative to a control IgG (Figure 3(e)), confirming the ability of hsa_circ_0000190 and miR-1253 to bind to one another within NSCLC cells. DDP-resistant NSCLC patient tissues also exhibited pronounced miR-1253 downregulation relative to DDP-sensitive tissues (Figure 3(f)), and hsa_circ_0000190 levels were negatively correlated with those of miR-1253 in tumor tissue samples from DDP-resistant patients (Figure 3(g)). The downregulation of miR-1253 was evident in parental NSCLC cells as compared to control HBE1 cells, while levels of this miRNA were further reduced in DDP-resistant NSCLC cells (Figure 3(h)). A pronounced increase in levels of hsa_circ_0000190 was evident in NSCLC cells when this circRNA was exogenously overexpressed (Figure 3(i)). When hsa_circ_0000190 was overexpressed, levels of miR-1253 fell markedly whereas the opposite was evident in A549/DDP and H460/DDP cells upon the suppression of hsa_circ_0000190 expression (Figure 3(j)). Overall, these results support a model in which hsa_circ_0000190 can act as a molecular sponge capable of sequestering miR-1253 within NSCLC cells.

### 3.4. Hsa_circ_0000190 Silencing Sensitizes NSCLC Cells to DDP in Part as a Result of miR-1253 Upregulation

**Figure 4:**
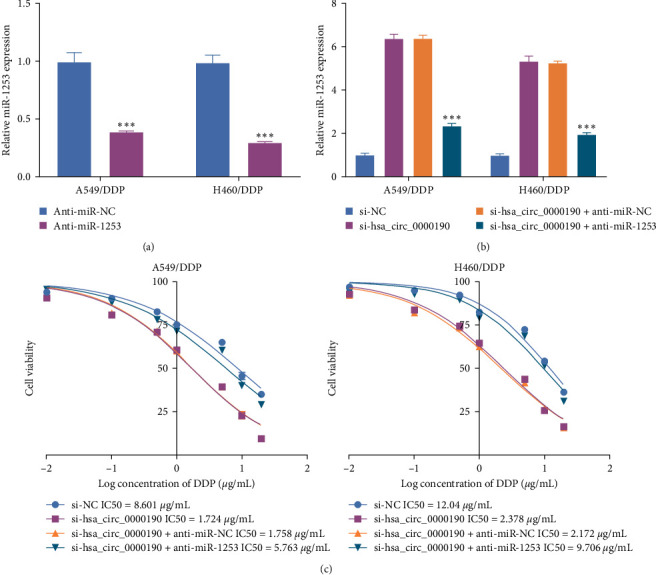
Hsa_circ_0000190 silencing sensitizes NSCLC cells to DDP in part as a result of miR-1253 upregulation. (a) qPCR was used to assess miR-1253 inhibitor efficiency. (b) Relative miR-1253 expression was analyzed via qPCR. (c) A CCK-8 assay was used to quantify DDP IC50 values.  ^*∗∗∗*^*P* < 0.001.

Given the observed negative regulatory association between hsa_circ_0000190 and miR-1253, a rescue experiment was conducted with the goal of validating this functional relationship and its implications for the ability of NSCLC cells to resist DDP treatment. The efficiency of miR-1253 inhibitor-mediator knockdown was confirmed via qPCR in these NSCLC cell lines (Figure 4(a)). Hsa_circ_0000190 knockdown resulted in miR-1253 upregulation in A549/DDP and H460/DDP cells, but the treatment of these cells with si-hsa_circ_0000190 and miR-1253 inhibitors was sufficient to rescue changes in the expression of this miRNA (Figure 4(b)). Notably, miR-1253 inhibitor transfection reversed the si-hsa_circ_0000190-associated changes in the chemoresistant status of A549/DDP and H460/DDP cells (Figure 4(c)). These data are consistent with a model in which the hsa_circ_0000190/miR-1253 axis controls the ability of NSCLC cells to resist DDP treatment.

### 3.5. IL-6 Is a miR-1253 Target mRNA

**Figure 5:**
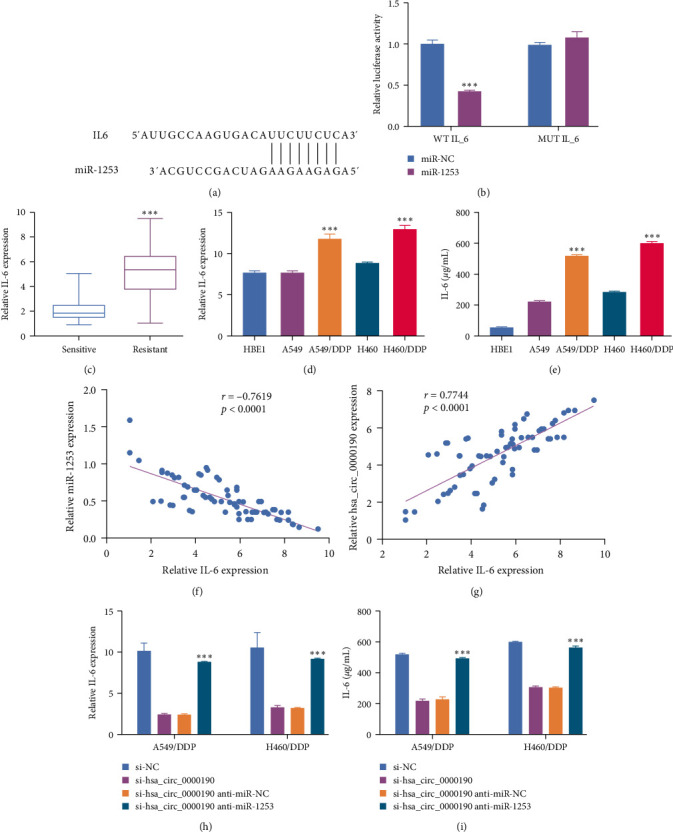
IL-6 is a miR-1253 target mRNA. (a) TargetScan identified Il-6 as a candidate miR-1253 target gene. (b) A dual-luciferase reporter assay confirmed the ability of miR-1253 to bind the IL-6 mRNA. (c) IL-6 levels were assessed via qPCR in NSCLC tumor tissues harvested from DDP-resistant (*n* = 73) and DDP-sensitive (*n* = 63) patients. (d, e). IL-6 levels were analyzed in NSCLC cell lines, DDP-resistant NSCLC cell lines, and control HBE1 cells via qPCR (d) and ELISA (e). (f, g) Spearman's correlation coefficients were used to assess relationships between IL-6 expression and the levels of miR-1253 or hsa_circ_0000190. (h, i) IL-6 levels were detected via qPCR and ELISA in NSCLC cells following transfection with si-NC, si-hsa_circ_0000190, si-hsa_circ_0000190 + miR-NC, or si-hsa_circ_0000190 + anti-miR-1253.  ^*∗∗∗*^*P* < 0.001.

The TargetScan tool identified IL-6 as a putative miR-1253 binding target (Figure 5(a)). Consistently, a dual-luciferase reporter assay demonstrated that miR-1253 expression suppressed the activity of a reporter bearing a WT but not a MUT version of the candidate miR-1253 binding sequence within the 3′ UTR of the IL-6 mRNA (Figure 5(b)), supporting the ability of these transcripts to interact within 293T cells. DDP-resistant NSCLC tissue samples also exhibited IL-6 mRNA levels significantly higher than those in DDP-sensitive tissues (Figure 5(c)). The expression of IL-6 in A549/DDP and H460/DDP cells were higher than those in the corresponding parental cells from which these sublines were derived (Figures 5(d) and 5(e)). In DDP-resistant NSCLC tissue samples, IL-6 mRNA expression was negatively correlated with the levels of miR-1253 but positively correlated with levels of hsa_circ_0000190 (Figures 5(f) and 5(g)). Silencing hsa_circ_0000190 resulted in pronounced IL-6 downregulation in both DDP-resistant cell lines, and this effect was counteracted by miR-1253 inhibitor treatment (Figures 5(h) and 5(i)), highlighting the ability of hsa_circ_0000190 to function as a molecular sponge for miR-1253 that promotes IL-6 upregulation in chemoresistant NSCLC cells.

### 3.6. MiR-1253 Suppresses IL-6 to Sensitize NSCLC Cells to DDP

**Figure 6:**
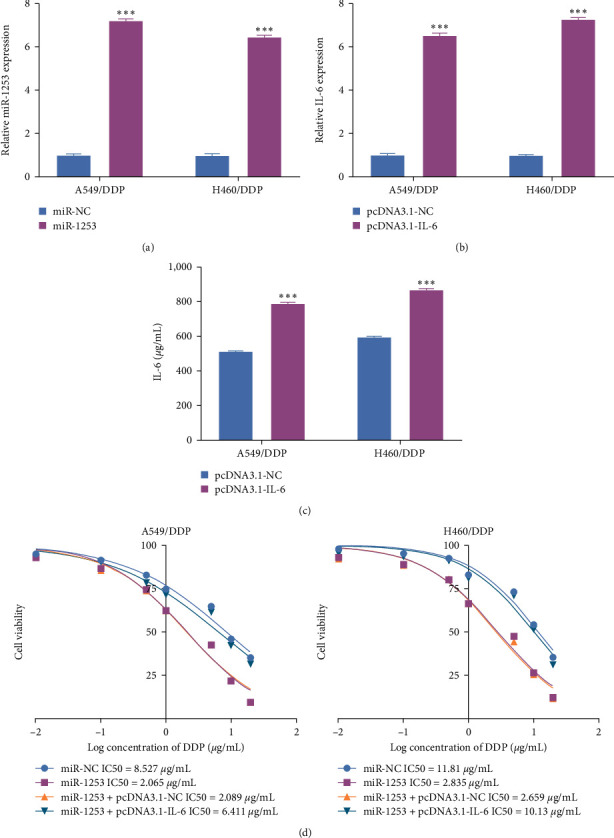
MiR-1253 suppresses IL-6 to sensitize NSCLC cells to DDP. (a) qPCR were used to assess miR-1253 expression. (b, c) qPCR and ELISA were used to assess IL-6 expression. (d) NSCLC cell resistance to DDP was assessed through a CCK-8 assay approach.  ^*∗∗∗*^*P* < 0.001.

To assess the ability of miR-1253 to modulate DDP resistance and NSCLC cell malignancy through the suppression of IL-6 expression, a final series of rescue assays was conducted. Both qPCR and ELISA approaches confirmed the efficiency of miR-1253 and pcDNA3.1-IL-6 transfection in DDP-resistant cells (Figure 6(a)–6(c)). The upregulation of miR-1253 sensitized these chemoresistant cells to DDP, whereas pcDNA3.1-IL-6 transfection partially restored DDP resistance to these cells as determined based on measured IC50 values (Figure 6(d)). These data suggest that miR-1253 can control NSCLC resistance to DDP in part through its ability to suppress IL-6 expression.

## 4. Discussion

High-throughput sequencing efforts have enabled the identification and characterization of a growing number of circRNAs [18, 19]. As they are rich in binding sites for miRNAs, circRNAs are often studied as miRNA sponges [20], providing a mechanism through which they control the onset and development of particular cancers [21]. In the present report, the ability of hsa_circ_0000190 to shape NSCLC resistance to DDP treatment was assessed in depth. Several prior publications have documented the importance of circRNAs as regulators of chemoresistant phenotypes. The circ-CPA4/let-7 miRNA/PD-L1 axis, for example, is reportedly capable of supporting NSCLC cell growth, chemoresistance, stemness, and the ability of these cells to evade immune-mediated elimination [22]. Exosomal circVMP1 can also facilitate NSCLC progression and resistance to DDP owing to its ability to modulate the miR-524-5 p-methyltransferase like 3 (METTL3)/sex determining region Y box 2 (SOX2) axis [23]. There is prior evidence for the ability of hsa_circ_0000190 to control NSCLC tumor progression [9]. In this study, the importance of hsa_circ_0000190 as a regulator of NSCLC cell DDP resistance was assessed. DDP-resistant NSCLC cell sublines exhibited increased hsa_circ_0000190 expression. When this circRNA was silenced in these DDP-resistant cells, this partially restored their chemosensitivity, emphasizing the status of hsa_circ_0000190 as a promoter of the ability of NSCLC cells to resist DDP treatment.

The most widely studied process through which circRNAs exert their biological functions is the so-called miRNA sponge mechanism [24]. Through, their ability to effectively sequester specific miRNAs, circRNAs can regulate the onset and progression of a range of cancer types [25]. The fact that hsa_circ_0000190 expression in NSCLC cells was primarily restricted to the cytosol was consistent with its ability to serve as a sponge for specific miRNAs. Consistently, predictive analyses identified miR-1253 as a candidate hsa_circ_0000190 binding target, and this was subsequently validated through RIP and luciferase reporter assays. DDP-resistant NSCLC cells and tissues exhibited pronounced miR-1253 downregulation. Moreover, there have been several prior studies documenting varied roles for miR-1253 in particular cancers. In NSCLC, for example, this miRNA has been reported to suppress proliferative activity and stem-like phenotypes [13]. Through its ability to target WNT5A, miR-1253 has also been reported to inhibit NSCLC invasivity and proliferation [15]. In this study, rescue experiments demonstrated that silencing hsa_circ_0000190 suppressed the resistance of NSCLC cells to DDP in part owing to the ability of this circRNA to act as a miR-1253 sponge.

A growing wealth of evidence has clearly demonstrated the ability of miRNAs to facilitate posttranslational target mRNA degradation [26]. The TargetScan database identified IL-6 as a putative miR-1253 target, as subsequently validated through a dual-luciferase reporter assay. IL-6 has been documented to enhance the chemoresistant properties of several types of cancers, for example through its ability to induce the phosphorylation of BECN1 and to control autophagic activity [17]. In colorectal cancer, HIF-1*α*/miR-338-5p/IL-6 axis activation in response to hypoxic conditions is also reportedly conducive to tumor growth [27]. The ability of cancer-associated fibroblasts to secrete IL-6 has also been mechanistically linked to the emergence of chemoresistant disease in individuals with NSCLC [28]. DDP-resistant tissue samples and cell lines exhibited high levels of IL-6 expression in the present analyses, and further research demonstrated that hsa_circ_0000190 was able to promote IL-6 upregulation through its ability to function as a sponge capable of sequestering miR-1253. Strikingly, interference with miR-1253 in NSCLC resistant cell lines was sufficient to enhance their resistance to DDP, at least in part owing to the consequent restoration of IL-6 expression.

In summary, DDP-resistant NSCLC cells and tissues exhibit pronounced hsa_circ_0000190 and IL-6 upregulation together with the downregulation of miR-1253. Functional assays demonstrated that hsa_circ_0000190 can target this miR-1253/IL-6 regulatory axis in a manner that promotes the emergence of DDP chemoresistance in NSCLC. Overall, these data highlight novel targets for efforts aimed at more effectively treating patients with NSCLC.

## Figures and Tables

**Table 1:** Relationship between hsa_circ_0000190 and clinicopathological data of NSCLC patients.

Clinical feature	Low hsa_circ_0000190	High hsa_circ_0000190	*P*
Age (years)			0.241
<55	25	21	
≥55	43	47	
Sex			0.185
Male	35	37	
Female	33	31	
TNM stage			<0.05
I–II	45	20	
III–IV	23	48	
Tumor size			<0.05
<5 cm	42	21	
≥5 cm	26	47	
Metastasis			0.091
No	33	31	
Yes	35	37	

## Data Availability

The data used to support the findings of this study are available from the corresponding author upon request.

## Supplementary Materials

Supplementary Materials Figure S1: a CCK-8 assay was used to quantify DDP IC50 values. Figure S2: qPCR was used to assess CNIH4 mRNA expression. Figure S3: a CCK-8 assay was used to quantify DDP IC50 values.
