# Supplementary material for: Hsa_circ_0000190 Promotes NSCLC Cell Resistance to Cisplatin via the Modulation of the miR-1253/IL-6 Axis
Source: Anal Cell Pathol (Amst). 2024 Feb 26;2024:6647810. doi: 10.1155/2024/6647810 (PMC10911877; doi:10.1155/2024/6647810)
Supplement: Supplementary Materials — Figure S1: a CCK-8 assay was used to quantify DDP IC50 values. Figure S2: qPCR was used to assess CNIH4 mRNA expression. Figure S3: a CCK-8 assay was used to quantify DDP IC50 values. [file 6647810.f1.docx]

**Supplementary materials**


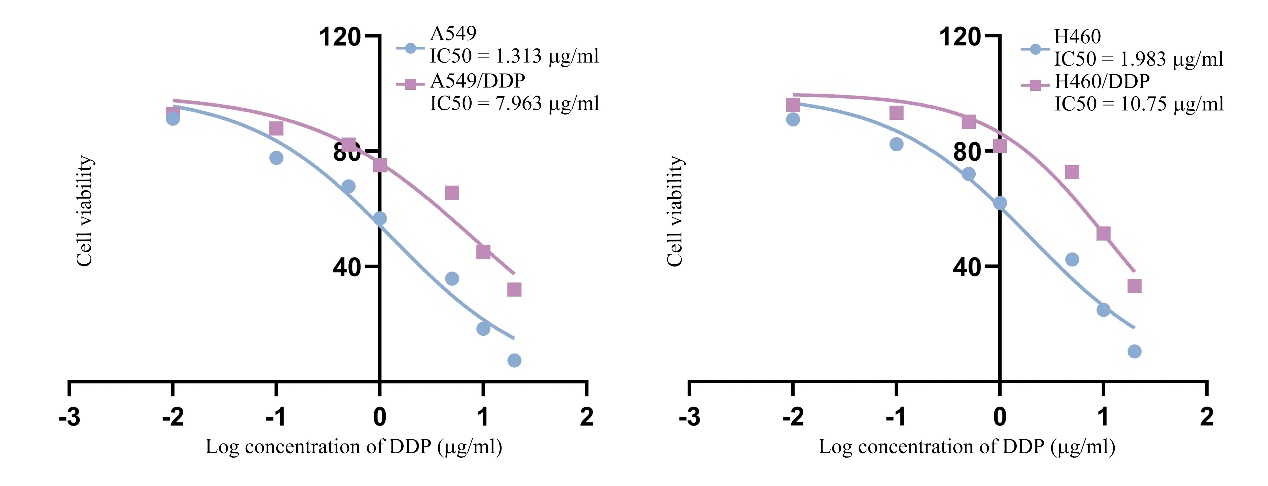


Figure S1. A CCK-8 assay was used to quantify DDP IC50 values.


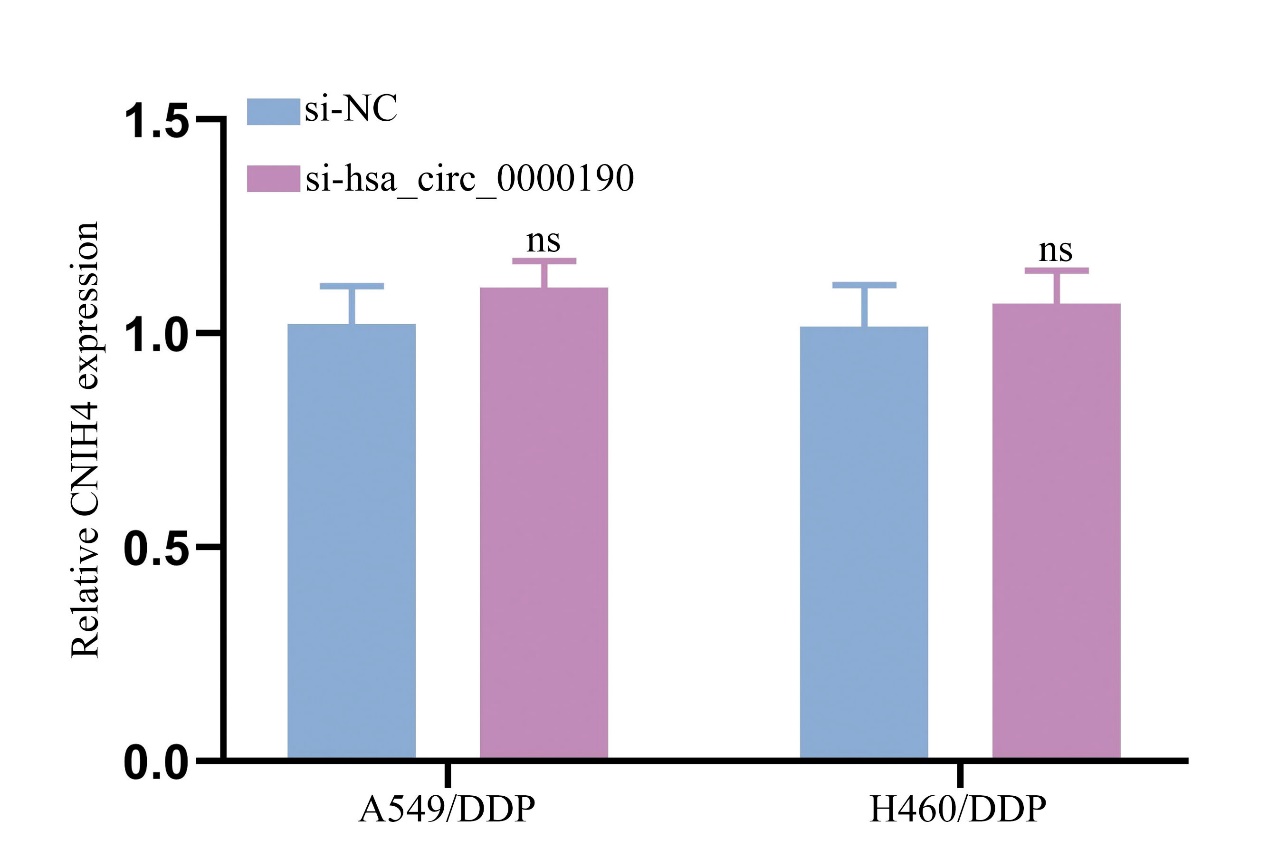


Figure S2. qPCR were used to assess CNIH4 mRNA expression.


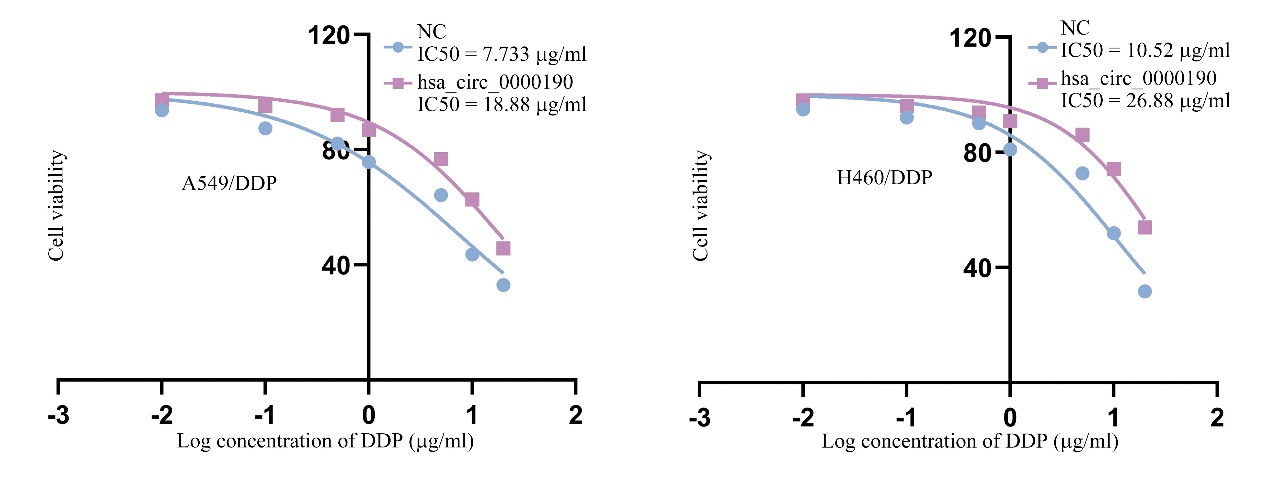


Figure S3. A CCK-8 assay was used to quantify DDP IC50 values.
